# The Effect of Nabilone on Neuropsychiatric Symptoms in Alzheimer's Disease: A secondary analysis

**DOI:** 10.1002/alz70857_105775

**Published:** 2025-12-24

**Authors:** Tiffany J. Pan, Hui Jue Wang, Nathan Herrmann, Myuri Ruthirakuhan, Damien Gallagher, Nicolaas Paul L.G. Verhoeff, Alex Kiss, Sandra E. Black, Krista L Lanctôt

**Affiliations:** ^1^ Hurvitz Brain Sciences Program, Sunnybrook Research Institute, Toronto, ON, Canada; ^2^ University of Toronto, Toronto, ON, Canada; ^3^ Baycrest Health Science Centre, Toronto, ON, Canada

## Abstract

**Background:**

Neuropsychiatric symptoms (NPS) are prominent in moderate‐severe Alzheimer's disease (AD), and are associated with poor quality of life, early institutionalization and caregiver distress. Nabilone showed promising results as an agitation treatment in the 14‐week Safety and Efficacy of Nabilone in Alzheimer's Disease trial, a randomized placebo‐controlled trial in moderate‐severe AD patients. We explored the treatment effects of nabilone on NPS other than agitation in the trial.

**Method:**

The trial included participants with Major Neurocognitive Disorder due to AD (Diagnostic and Statistical Manual‐5 criteria), scored ≤24 on the Standardized Mini‐Mental State Examination, and had clinically significant agitation (Neuropsychiatric Inventory‐Nursing Home version (NPI‐NH)‐Agitation/Aggression (A/A) ≥3). It was a randomized crossover trial with 6‐week treatment phases for nabilone and placebo, and a 1‐week placebo washout period. NPI‐NH was assessed at the start and the end of each treatment phase. Linear mixed models were fitted to compare the between‐treatment phases for the change in each non‐agitation NPI‐NH sub‐scale, except delusions/hallucinations, which was a trial exclusion criterion. The models included treatments (2 levels: nabilone and placebo) and baseline scores as fixed factors, and a random factor of participant code accounting for individual variability.

**Result:**

Thirty‐nine participants with agitation (mean (SD) NPI‐NH‐A/A: 7.0 (3.2)) were recruited (77% male, age: 87 (10), nabilone dose: 1.6 (0.5) mg orally daily). There were greater decreases in anxiety (b=‐1.12, SE=0.55, *p* = 0.048) and depression (b=‐0.68, SE=0.31, *p* = 0.039) and greater changes in appetite/eating habits (b=2.00, SE=0.55, *p* < .001) observed during nabilone compared to placebo. Baseline scores (BL) for anxiety (BL nabilone/placebo: 4.6 (4.6)/4.6 (4.5), b=‐0.32, SE=0.08, *p* < .001), depression (BL nabilone/placebo: 1.0 (3.0)/2.0 (3.0), b=‐0.15, SE=0.06, *p* = 0.021), and appetite/eating habits (BL nabilone/placebo: 2.8 (3.7)/2.5 (3.6), b=‐0.32, SE=0.08, *p* < .001) were significant predictors of the change in respective sub‐scale scores, with higher baseline scores predicting a greater score decrease in treatment phases.

**Conclusion:**

In addition to the efficacy for improving agitation shown previously, these exploratory results suggest that nabilone can also alleviate anxiety and depression symptoms in moderate‐severe AD patients. The change in appetite/eating habits required further attention, as the specific changes (increase or decrease in appetite) were unclear.

